# ACADEMIC-NGO PARTNERSHIP IN CONSTRUCTING A LOGIC MODEL FOR EVALUATION OF A CARER-CENTRIC COMMUNITY-BASED PROJECT

**DOI:** 10.1093/geroni/igae098.3337

**Published:** 2024-12-31

**Authors:** Vera Mun Yu Tang, Vivian Weiqun Lou, Tarani Chandola, Clio Yuen Man Cheng, Iris Xiaoyu Ruan, Vincy Wing Sze Pang

**Affiliations:** The University of Hong Kong, Hong Kong, China (People’s Republic); The University of Hong Kong, Hong Kong, China (People’s Republic); The University of Hong Kong, Hong Kong, China (People’s Republic); The University of Hong Kong, Hong Kong, China (People’s Republic); The University of Hong Kong, Hong Kong, China (People’s Republic); The University of Hong Kong, Hong Kong, China (People’s Republic)

## Abstract

Evaluation is the cornerstone of demonstrating a particular project’s success and paving the way for continuous improvement. However, most of the existing evaluation in social service sector focused on outcome, whereas some evaluated process and a very few included both process and outcome evaluation. Logic models have been found useful in programme evaluation but little is known about how to construct and apply logic models for guiding the process and outcome evaluation of community-based projects, and even less for projects with academic-NGO partnership. A pilot project adopting a carer-centric approach has been launched in 2023, which is anticipated to serve carers and older adults with caring needs in the district-based caregiver support units operated by five NGO partners in Hong Kong. This paper reports how the university as a project partner constructs a comprehensive logic model for evaluation through engaging different stakeholders (including NGO partners, funder, and potential beneficiaries) in two rounds of small group semi-structured interview. Deductive approach is applied for thematic analysis using MAXQDA to identify and prioritize inputs, activities, outputs, and outcomes of the project at initiate phase. Findings provide academics, NGOs and funding bodies with fresh insights into new elements of logic model, including volunteers as important inputs, co-creation workshops as important activities for project positioning and service alignment, number of referral cases as important outputs, and experience consolidation for service adjustment as important intermediate outcomes. This study highlights that process and outcome evaluation can be advanced by promoting stakeholder engagement to co-construct logic model.

